# Supplementary material for: Conyza canadensis from Jordan: Phytochemical Profiling, Antioxidant, and Antimicrobial Activity Evaluation
Source: Molecules. 2024 May 20;29(10):2403. doi: 10.3390/molecules29102403 (PMC11123705; doi:10.3390/molecules29102403)
Supplement: Supplementary file 1 [file molecules-29-02403-s001.zip › molecules-2978049-supplementary.pdf]

# Supplementary

# *Conyza canadensis* from Jordan: Phytochemical Profiling, Antioxidant, and Antimicrobial Activity Evaluation

**Lina M. Barhoumi [1,*], Ashok K. Shakya[2,*], O'la Al-Fawares[3] and Hala I. Al-Jaber [1]**

[1] Chemistry Department, Faculty of Science, Al-Balqa Applied University, Al-Salt 19117, Jordan; hala.aljaber@bau.edu.jo

[2] Pharmacological and Diagnostic Research Center, Faculty of Pharmacy, Al-Ahliyya Amman University, Amman 19328, Jordan

[3] Medical Laboratory Sciences Department, Faculty of Science, Al-Balqa Applied University, Al-Salt 19117, Jordan; ola.alfawares@bau.edu.jo

* Correspondence: linabrhoumi@bau.edu.jo (L.M.B.); ak_shakya@ammanu.edu.jo (A.K.S.)

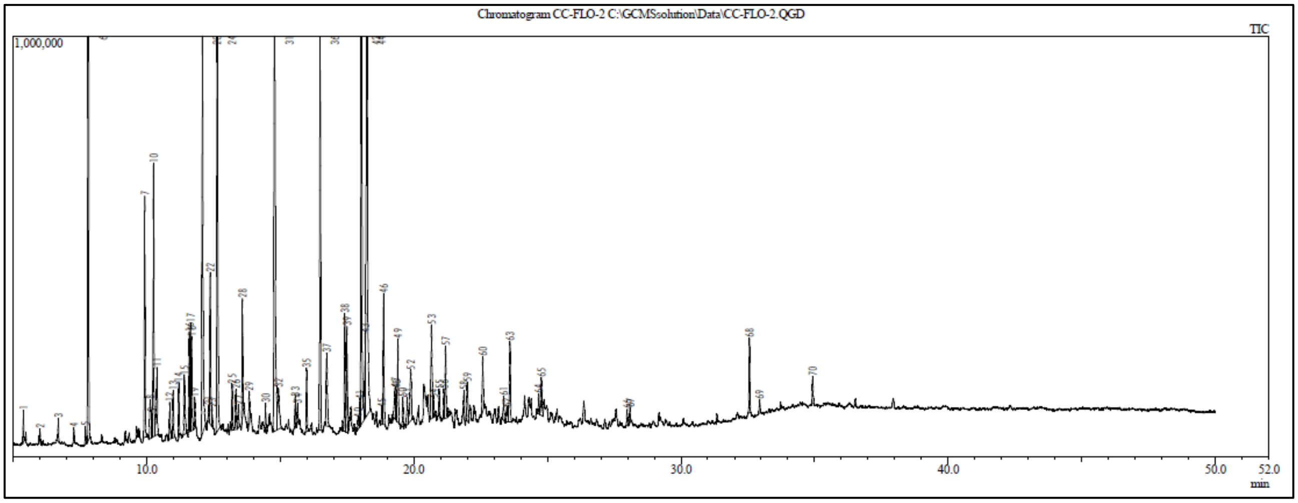


**Figure S1** GC-MS for CCHD-EO of inflorescence heads

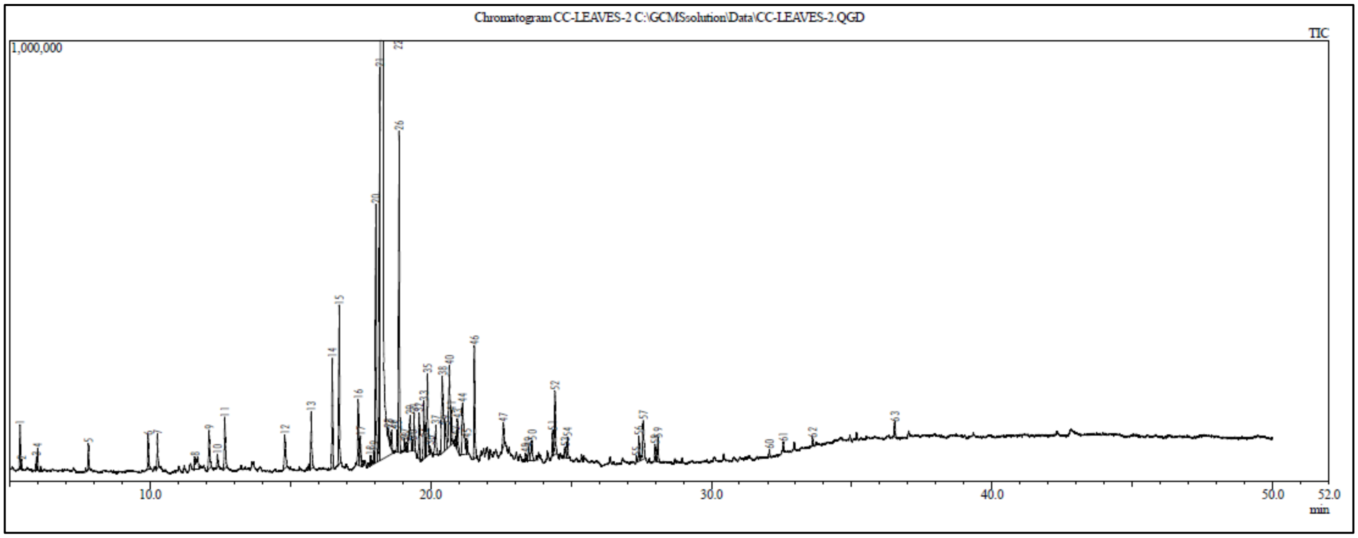


**Figure S2** GC-MS for CCHD-EO of leaves

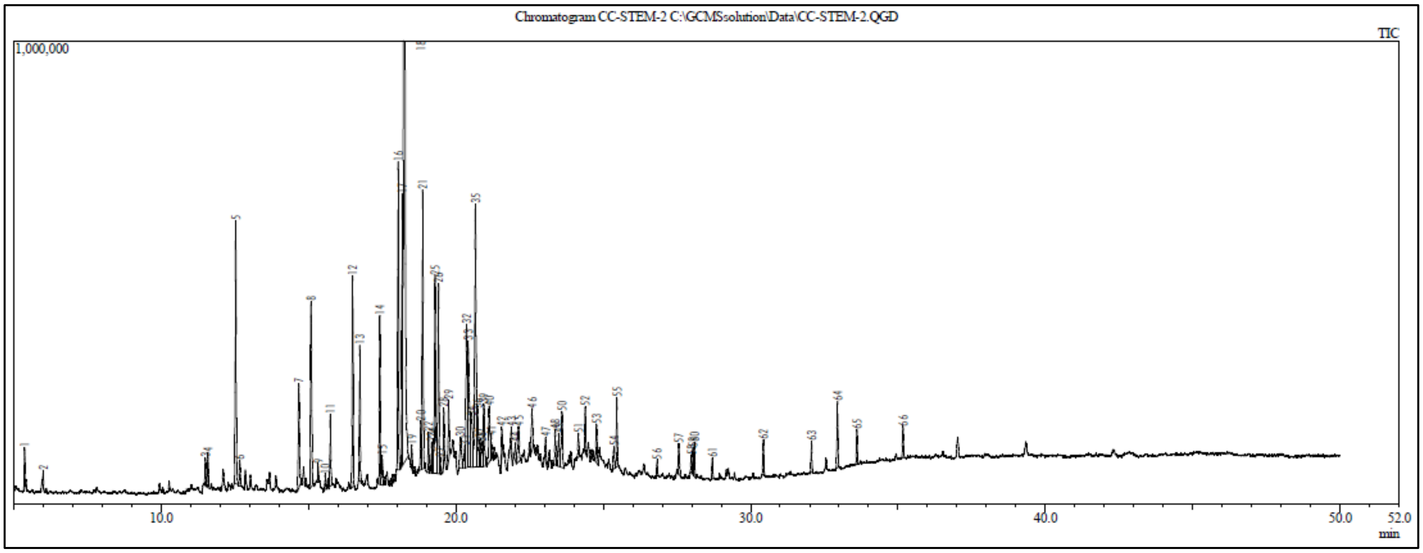


**Figure S3** GC-MS for CCHD-EO of stems.

**Table S1** The LOD, LOQ, range, fragments and supplier for the standard phenolic compounds and flavonoids used in LC-MS/MS analysis

| No. | Compound name | Molecular Formula | Structural formula | $R_t$ (min) | LOD(LOQ) ng/mL | Range ng/mL | Fragments | Supplier |
|---|---|---|---|---|---|---|---|---|
| **Phenolic Compounds** | | | | | | | | |
| 1. | Vanillic acid | $C_8H_8O_4$ | 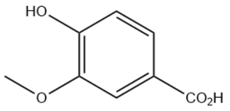  | 3.217 | 100 (300) | 300-8000 | 166.828/107.80 | MilliporeSigma, (USA) |
| 2. | Syringic acid | $C_9H_{10}O_5$ | 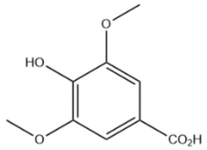  | 3.598 | 100 (300) | 300-8000 | 196.912/152.80 | Biosynth (UK) |
| 3. | *p*-Coumaric acid | $C_9H_8O_3$ | 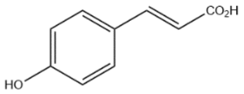  | 4.449 | 100 (300) | 300-8000 | 163.221/118.80 | Biosynth (UK) |
| 4. | Ferulic acid | $C_{10}H_{10}O_4$ | 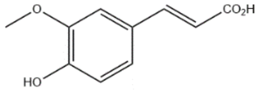  | 4.800 | 100 (300) | 300-8000 | 192.766/133.80 | Biosynth (UK) |
| 5. | Resveratrol | $C_{14}H_{12}O_3$ | 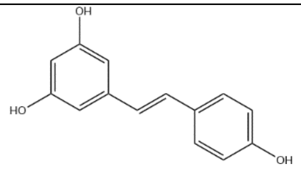  | 5.561 | 100 (300) | 300-8000 | 226.946/184.80 | MilliporeSigma, (USA) |

| | | | | | | | | |
|---|---|---|---|---|---|---|---|---|
| 6. | Rosmarinic acid | $C_{18}H_{16}O_8$ | 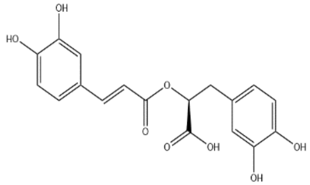 | 5.863 | 100 (300) | 300-8000 | 358.782/160.80 | Extrasynthese, (France) |
| 7. | Salvianolic acid B | $C_{36}H_{30}O_{16}$ | 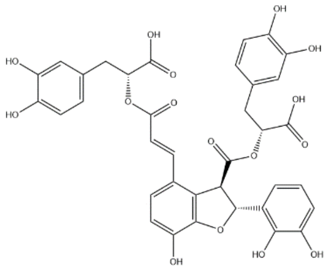 | 6.151 | 100 (300) | 300-8000 | 716.709/518.70 | Extrasynthese, (France) |
| 8. | Salvianolic acid A | $C_{26}H_{22}O_{10}$ | 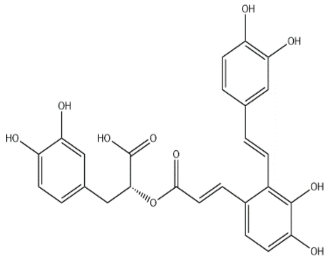 | 6.948 | 100 (300) | 300-8000 | 492.732/294.70 | Extrasynthese, (France) |
| 9. | Chlorogenic acid | $C_{16}H_{18}O_9$ | 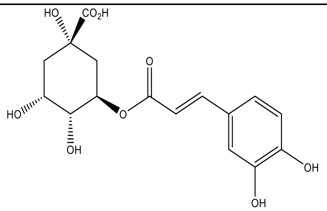 | 7.560 | 100 (300) | 300-8000 | *252.877/96.90* | Sigma-Aldrich(USA) |

| | | | | | | | | |
|---|---|---|---|---|---|---|---|---|
| 10. | Caffeic acid phenethyl ester (CAPE) | $C_{17}H_{16}O_4$ | 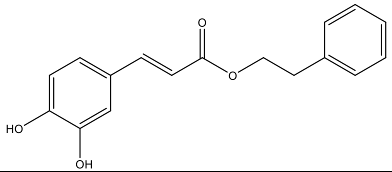 | 9.215 | 100 (300) | 300-8000 | 282.845/178.600 | Sigma-Aldrich(USA) |
| 11. | Gallic acid | $C_7H_6O_5$ | 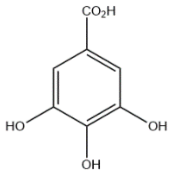 | 9.467 | 100 (300) | 300-8000 | 168.904/124.800 | Sigma-Aldrich(USA) |
| 12. | Carnosic acid | $C_{20}H_{28}O_4$ | 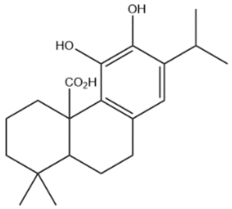 | 20.693 | 100 (300) | 300-8000 | 330.989/286.800 | Extrasynthese, (France) |
| **Flavonoids** | | | | | | | | |
| 1. | Catechin | $C_{15}H_{14}O_6$ | 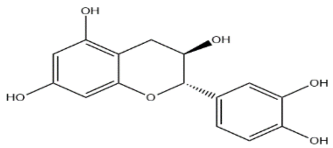 | 2.005 | 100 (300) | 300-8000 | 288.837/244.600 | MilliporeSigma (USA) |
| 2. | Hesperidin | $C_{28}H_{34}O_{15}$ | 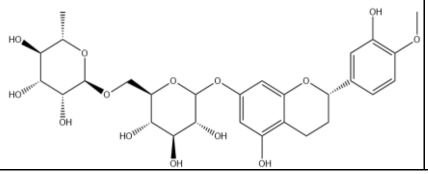 | 5.599 | 100 (300) | 300-8000 | 608.785/300.70, 608.785/163.600 | MilliporeSigma (USA) |

| | | | | | | | | |
|---|---|---|---|---|---|---|---|---|
| 3. | Rutin | $C_{27}H_{30}O_{16}$ | 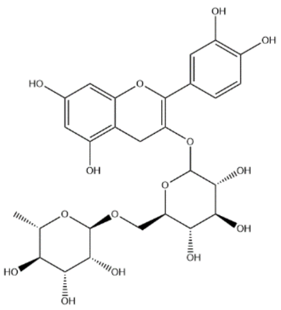 | 5.856 | 100 (300) | 300-8000 | 608.799/299.700 | MilliporeSigma (USA) |
| 4. | Apigenin-7-O-glucoside | $C_{21}H_{20}O_{10}$ | 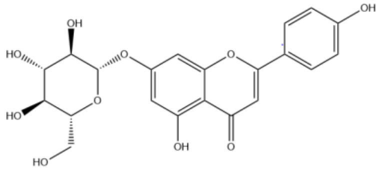 | 6.172 | 100 (300) | 300-8000 | 430.757/267.700 | Extrasynthese, (France) |
| 5. | Hesperetin | $C_{16}H_{14}O_{6}$ | 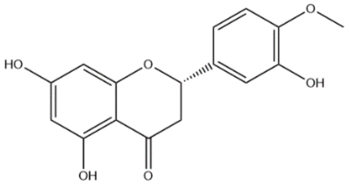 | 6.992 | 100 (300) | 300-8000 | 300.746/163.700 | MilliporeSigma (USA) |
| 6. | Quercetin | $C_{15}H_{10}O_{7}$ | 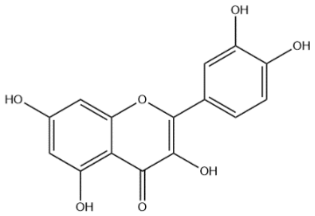 | 7.194 | 100 (300) | 300-8000 | 300.725/150.800 | Sigma-Aldrich (USA) |

| | | | | | | | | |
|---|---|---|---|---|---|---|---|---|
| 7. | Luteolin | $C_{15}H_{10}O_6$ | 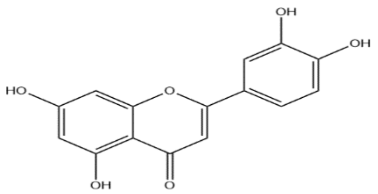 | 7.265 | 100 (300) | 300-8000 | 284.726/132.800 | Extrasynthese, (France |
| 8. | Apigenin | $C_{15}H_{10}O_5$ | 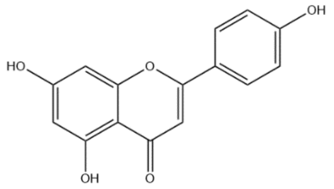 | 8.097 | 100 (300) | 300-8000 | 268.810/116.900 | Extrasynthese, (France |
| 9. | 3-O-Methylquercetin | $C_{16}H_{12}O_7$ | 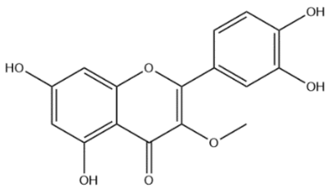 | 8.031 | 100 (300) | 300-8000 | 314.404/299.600 | MilliporeSigma (USA |

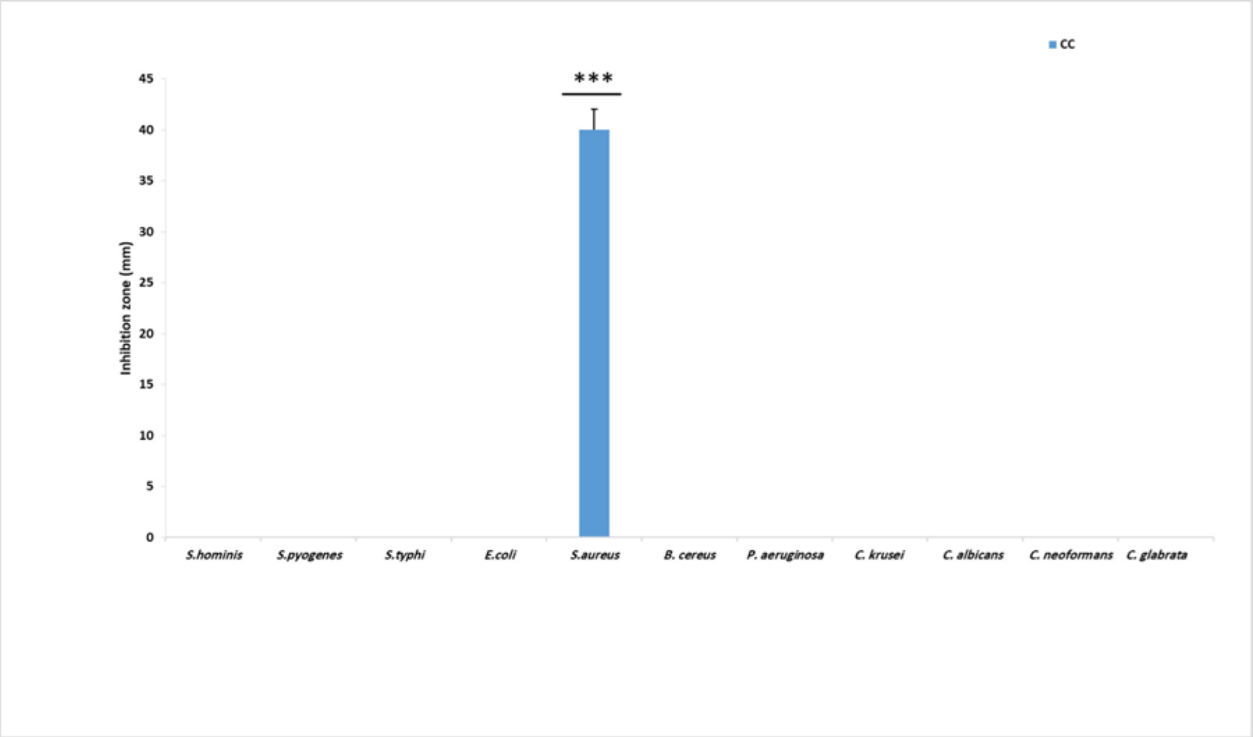


**Figure S4** Zone of inhibition bar graph introduced by CCM against studied microorganisms.

***The asterisks indicate a significance of P< 0.001
